# Association of obesity indicators with cognitive function among US adults aged 60 years and older: Results from NHANES

**DOI:** 10.1002/brb3.70006

**Published:** 2024-09-11

**Authors:** Leian Chen, Ying Hou, Yu Sun, Dantao Peng

**Affiliations:** ^1^ China‐Japan Friendship Hospital (Institute of Clinical Medical Sciences) Chinese Academy of Medical Sciences & Peking Union Medical College Beijing China; ^2^ Department of Neurology China‐Japan Friendship Hospital Beijing China; ^3^ Peking University China‐Japan Friendship School of Clinical Medicine Beijing China

**Keywords:** cognitive function, obesity, older adult, structural equation modeling

## Abstract

**Background:**

Midlife obesity is a significant risk factor for Alzheimer's disease, but the effects of obesity on cognitive function, either detrimental or beneficial, are controversial among older individuals. This study aims to assess this associations of body mass index (BMI) or waist circumference (WC) with cognitive function among United States older individuals.

**Methods:**

A cross‐sectional research study was conducted utilizing data from the 2011 to 2014 National Health and Nutrition Examination Survey (NHANES). Initially, the study compared differences in cognitive function among the normal weight, overweight, and obese groups. Subsequently, we examined the relationships between BMI or WC and cognitive function using multivariate linear regression. Finally, structural equation models were constructed to assess the relationships among body shape, lifestyle, and cognitive function pathways.

**Results:**

The study included 2254 individuals. Obese subjects had lower scores in the Consortium to Establish a Registry for Alzheimer's Disease (CERAD) word list learning tasks (CERAD‐WL) (*χ* ^2^ = 7.804, *p* = .020) and digit symbol substitution test (*χ* ^2^ = 8.869, *p* = .012). The regression analysis showed that WC was negatively connected with the CERAD‐WL score after adjusting for confounding factors (*β* = −.029, *p* = .045). Moreover, WC had a mediating effect on the path from lifestyle to cognition (CERAD‐WL). However, there was no difference in the CERAD delayed recall score and the animal fluency test between the obese and the other groups.

**Conclusions:**

Obese older adults exhibited impaired cognitive abilities in terms of learning and working memory performance. The impact of lifestyle on cognition was mediated by obesity‐related anthropometric indices. Sleep, physical activity, and diet influenced the degree of obesity, which subsequently determined cognitive function. Prioritizing weight management in elderly people is crucial for safeguarding cognitive function.

## Graphical abstract

Obese subjects had lower scores in the CERAD word list learning tasks (CERAD‐WL) and digit symbol substitution test (DSST). Body shape had a mediating effect on the path from lifestyle to cognition (CERAD‐WL). Sleep, physical activity, and diet influenced the degree of obesity, which subsequently determined cognitive function.

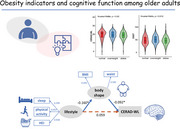

## 1 INTRODUCTION

With the progressive aging of the population, there has been a concurrent increase in the prevalence of obesity in the elderly population (Hales et al., 2020). Obesity in older patients is a significant concern due to its detrimental physical and psychosocial effects (Vaidya, 2006). It should be noted that obesity is not solely characterized by metabolic changes but rather manifests as a multisystem disease. It impacts nearly every organ system, including the endocrine, gastrointestinal, cardiovascular, and musculoskeletal systems (Tsai & Bessesen, 2019). In addition to metabolic alterations, obesity is also linked to modifications in the central nervous system (Dye et al., 2017). Adiposity causes several poor cognition and neurocognitive changes (Morys et al., 2021; Mina et al., 2023). A study by Morys et al. (2021) revealed that obesity was related to worse fluid intelligence and working memory (WM). Increased body mass index (BMI), waist circumference (WC), and waist–hip ratio (WHR) were independently positively correlated with the incidence of cognitive impairment in adults older than 65 years (Liu et al., 2019). As a modifiable factor, midlife obesity is regarded as a risk factor for Alzheimer's disease (AD) and vascular dementia (Dye et al., 2017; Lisko et al., 2021; Zhang et al., 2021). Previous research has shown that obese individuals have a higher incidence of dementia diagnosis in later stages of life (Floud et al., 2020). On the other hand, obesity status is also associated with structural brain abnormalities, including larger white matter hyperintensities (Caunca et al., 2019), lower gray matter volume, more lacunar infarcts, and cerebral microbleeds (Han et al., 2021). Many biological mechanisms could explain the link between obesity and neuronal injury. Central inflammation caused by excess adipos alters synaptic plasticity and even initiates brain atrophy (Ellulu et al., 2017; Miller & Spencer, 2014). Other mechanisms may involve insulin/leptin resistance (Ahmed et al., 2021), endothelial dysfunction (Buie et al., 2019), and gut dysbiosis (Thornton et al., 2024). Moreover, individuals with obesity and individuals with AD share spatially similar cortical atrophy patterns and abnormal protein distributions (Morys et al., 2023). This adds to the previous findings about the influence of obesity on brain structure and function.

However, the link between late‐life obesity and cognition is inconclusive due to the varying impact of obesity on dementia across different stages of life (Puzianowska‐Kuznicka et al., 2019, 2022; Ren et al., 2021; Shinohara et al., 2023). Several studies have suggested that, compared with a normal BMI, obesity in late life reduces the risk of dementia (Shinohara et al., 2023). Conversely, a study based on older Chinese adults found that the protective effect of overweight/obesity on cognitive function disappeared after adjusting for confounding factors (Ren et al., 2021). Similarly, another study concluded that overweight and obesity in Polish Caucasian seniors were not associated with the deterioration of cognitive function (Puzianowska‐Kuznicka et al., 2019). Given these inconsistent findings, an up‐to‐date understanding of the association between obesity and anthropometric indices is needed, which would help to refine strategies for the maintenance of cognitive function.

It is widely known that an unhealthy lifestyle is a major cause of obesity, so lifestyle modifications are consistently advocated as the primary therapy for obesity (Kheniser et al., 2021). Moreover, unhealthy lifestyle factors not only contribute to obesity but are also considered to be significant risk factors for cognitive impairment (Scheltens et al., 2021). In contrast, maintaining a healthy lifestyle has been linked to a reduced risk of cognitive decline and AD (Zhang et al., 2021). Sleep status, physical activity (PA), and diet are three main aspects of lifestyle, and maintaining daily healthy lifestyles is helpful for improving cognition (De La Rosa et al., 2020; Zhang et al., 2021; Zhao et al., 2018). BMI and WC are two globally recognized indicators for defining the degree of obesity and the nutritional status of the human body (Cornier et al., 2011). This study aims to describe the cognitive function of different domains among older adults with varying BMI and WC statuses, to assess the complex links among obesity, lifestyle factors, and poor cognition using data from the National Health and Nutrition Examination Survey (NHANES).

## 2 METHODS

### 2.1 Study design and participants

**FIGURE 1:**
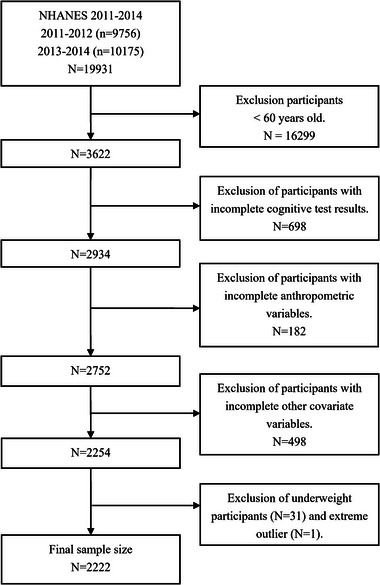
Flowchart of the selection of participants from the National Health and Nutrition Examination Survey (NHANES) 2011–2014.

The NHANES, a publicly available program conducted by the Centers for Disease Control and Prevention (CDC), is designed to assess the health and nutritional status of the civilian population in the United States. All NHANES protocols were approved by the CDC's National Center for Health Statistics Ethics Review Board, with every participant providing written informed consent before participation. This study conducted a cross‐sectional analysis using the data obtained from the 2011 to 2014 NHANES because only these two cycles (2011–2012, 2013–2014) covered the cognitive performance results. A total of 19,931 subjects were enrolled in these two stages of the investigation. This study included participants aged 60 years or older (*n* = 3622), as cognitive assessments in the NHANES study were conducted only on this age group. We extracted data on obesity indicators (BMI and WC), cognitive function, PA, eating habits, sleep, chronic disease status, and sociodemographic information from the NHANES. After excluding subjects without complete data, 2254 participants remained in our present study. Finally, all included variables were aggregated into a single dataset for subsequent analysis. The specific screening process is shown in Figure 1.

### 2.2 Obesity indicators

In this study, the dependent variable was BMI or WC. BMI was calculated as weight in kilograms divided by the square of height in meters. According to the cutoff thresholds recommended by the WHO, participants were categorized into three groups: normal weight (18.5 ≤ BMI < 25.0 kg/m^2^), overweight (25.0 ≤ BMI < 30.0 kg/m^2^), and obese (BMI ≥ 30.0 kg/m^2^) (Brandkvist et al., 2019). Underweight participants (BMI < 18.5 kg/m^2^) were excluded due to low prevalence (*N* = 31 (1.3%)).

### 2.3 Cognitive function assessment

Participants underwent a series of cognitive assessments, including word learning and recall modules from the Consortium to Establish a Registry for AD (CERAD), the animal fluency test (AFT), and the digit symbol substitution test (DSST). In this study, the score of the word list learning task of the CERAD (CERAD‐WL) was the sum of the correct words in three learning trials, and the score of the recall task of the CERAD (CERAD‐DR) was the number of correct memory words in the recall tasks. The CERAD‐WL examines immediate memory, whereas the CERAD‐DR evaluates delayed memory (Fillenbaum & Mohs, 2023). Immediate and delayed memories are two aspects of memory capability. Immediate memory, considered the inaugural stage of short‐term memory, primarily focuses on the immediacy and fleeting nature of information, whereas delayed memory involves more intricate cognitive processes, including the consolidation, storage, and retrieval of memories (Belanoff et al., 2001). The AFT was designed to assess executive function and language ability, with the total number of successfully named animals in 1 min as the final score (Whiteside et al., 2016). The DSST reflects processing speed, sustained attention, and WM (Brody et al., 2019).

### 2.4 Other important variables: lifestyle factors

There are three aspects of lifestyle factors in this study, namely, PA, sleep, and eating habits. The PA information was obtained through self‐reports, utilizing the Global Physical Activity Questionnaire. Minutes per week spent in different domains of PA (leisure‐time activity, work‐related activity, and transportation) were calculated. Each activity was assigned a metabolic equivalent (MET) score as recommended by NHANES. An MET is an indicator of relative energy metabolism and exercise intensity, which quantifies the level of energy expenditure during a specific PA relative to resting metabolic (quiet sitting) states (Mendes et al., 2018). The MET score for “vigorous work‐related activity” or “vigorous leisure‐time PA” is 8, and the MET score for “moderate work‐related activity,” “walking or bicycling for transportation,” or “moderate leisure‐time PA” is 4. We computed the cumulative MET‐minutes per week for each activity and derived a total weekly MET‐minute score of moderate and vigorous activities for every participant (Farag et al., 2023; Wanner et al., 2017).

We calculated sleep pattern scores based on sleep duration and the presence of trouble sleeping. Sleep duration data were collected using self‐report questionnaires (SLD010H). Sleep duration was further classified in this study as short (≤6 h per night), normal (7–9 h per night), or long (>9 h per night) based on previous studies. A sleep duration of 7–9 h per night was assigned a value of 1, whereas shorter or longer durations were assigned a value of 0. Trouble sleeping was measured by the specific question (SLQ050): “Have you ever told a doctor or other health professional that you have trouble sleeping?” If the participant answered no, the component of trouble sleeping was assigned as 1. The overall sleep pattern scores were generated by adding the sleep duration and trouble sleeping scores, which ranged from 0 (poor) to 2 (healthy) (Chen et al., 2023).

The dietary information was derived from the NHANES dietary interview and the Food Patterns Equivalents Database files. Only the first 24‐h dietary intake in the NHANES interview was chosen for analysis in this study (Jayanama et al., 2021). We used the Healthy Eating Index (HEI)‐2020 to evaluate eating habits (Krebs‐Smith et al., 2018; Shams‐White et al., 2023). A higher score reflects healthier eating habits. The scoring components for the HEI‐2020 are provided in Table S1. HEI‐2020 was calculated by the R package “Dietaryindex” (Zhan et al., 2023).

### 2.5 Statistical analysis

Given the complicated survey design of NHANES studies, specific sample weights were taken into consideration following NHANES analytic standards. Continuous variables with a normal distribution are reported as mean ± standard deviation, and one‐way ANOVA was used for comparisons between groups. Normally distributed data are expressed as medians and interquartile distances [M (Q1, Q3)], and the Kruskal–Wallis *H* test was used for comparisons between groups. The categorical variables are presented as percentages, and comparisons between groups were conducted with the Pearson *χ* ^2^ test. For multiple comparisons, the Scheffe method was used for normally distributed measurement data, the Nemenyi method was used for nonnormally distributed measurement data, the Bonferroni method was used for categorical data without order, and the Nemenyi method was used for categorical data with order.

Propensity score matching (PSM) was used to eliminate bias and control for potential confounding variables. In this study, using the 1:1:1 nearest neighbor matching algorithm without putting back, PSM was used to match normal weight, overweight, and obese participants. Confounding factors, including age, sex, race, education, smoking status, and alcohol consumption, were chosen for matching. PSM was conducted on an online website based on R software (https://mengte.online). Weighted analysis was performed due to the complicated survey design of the NHANES using specific sample weights. Multivariate linear regression analysis was used to evaluate the correlation between obesity indicators (BMI or WC) and cognitive function in different models. Model 1: Confounding variables were not adjusted for. Model 2: Age, sex, race, and education were adjusted for. Model 3 was adjusted for age, sex, race, PIR, education, smoking status, alcohol consumption, diabetes status, hyperlipidemia status, hypertension status, stroke history, and coronary artery disease.

Finally, we constructed a structural equation model (SEM) to determine complex associations among lifestyle, body shape, and cognition. Partial least squares SEM (PLS‐SEM) was performed to validate the measurements and test the proposed hypotheses. Sleep score, PA, and HEI2020 score were manifest variables, and lifestyle, body shape, disease situation, and cognition were latent variables. We hypothesized that body weight served as a mediating variable, mediating the relationship between lifestyle and cognitive performance directly or indirectly. PLS‐SEM is a causal‐predictive approach to SEM, which enables researchers to estimate complex models with many constructs, indicator variables, and structural paths without imposing distributional assumptions on the data (Hair et al., 2019). PLS‐SEM allows the assessment of interconstruct relationships as well as relationships among constructs and their respective indicators (Sun et al., 2023). All the statistical analyses except PSM were conducted using R (version 4.1.2, http://www.R‐project.org). A two‐tailed *p* value <.05 was considered to indicate statistical significance.

## 3 RESULTS

### 3.1 Baseline characteristics of participants

We selected 2222 participants who had complete information on both cognitive performance and covariates among 19,931 subjects. The characteristics of the participants are shown in Table 1. By applying sampling weights and stratification variables, these 2222 seniors represented 43,115,808 older people in this age range in the United States. The survey‐weighted characteristics of the sample are shown in Table S2.

**TABLE 1:** Characteristics of participants in the 2011–2014 National Health and Nutrition Examination Survey (NHANES).

Characteristic	Total (*n *= 2222)	Normal (*n *= 556)	Overweight (*n *= 787)	Obese (*n *= 879)	*p* Value
**Age, Mean ± SD, years**	69.3 ± 6.7	70.1 ± 6.8	69.8 ± 6.9	68.3 ± 6.4	<.001
**Sex, *n* (%)**					<.001
Male	1070 (48.2)	264 (47.5)	441 (56.0)	365 (41.5)	
Female	1152 (51.8)	292 (52.5)	346 (44.0)	514 (58.5)	
**Race, *n* (%)**					<.001
Mexican American	184 (8.3)	28 (5.0)	71 (9.0)	85 (9.7)	
Other Hispanic	207 (9.3)	44 (7.9)	86 (10.9)	77 (8.8)	
Non‐Hispanic White	1141 (51.4)	288 (51.8)	427 (54.3)	426 (48.5)	
Non‐Hispanic Black	516 (23.2)	106 (19.1)	143 (18.2)	267 (30.4)	
Non‐Hispanic Asian	144 (6.5)	82 (14.7)	49 (6.2)	13 (1.5)	
Other/multiracial	30 (1.4)	8 (1.4)	11 (1.4)	11 (1.3)	
**Education level, *n* (%)**					.002
Less than 9th grade	209 (9.4)	56 (10.1)	66 (8.4)	87 (9.9)	
9–11th grade (12th grade with no diploma)	292 (13.1)	62 (11.2)	106 (13.5)	124 (14.1)	
High school graduate/GED	524 (23.6)	124 (22.3)	183 (23.3)	217 (24.7)	
Some college or AA degree	656 (29.5)	143 (25.7)	227 (28.8)	286 (32.5)	
College graduate or above	541 (24.3)	171 (30.8)	205 (26.0)	165 (18.8)	
**PIR, Mean ± SD**	2.7 ± 1.6	2.8 ± 1.6	2.8 ± 1.6	2.5 ± 1.6	.006
**Smoke, *n* (%)**					<.001
Never	1092 (49.1)	274 (49.3)	379 (48.2)	439 (49.9)	
Former	875 (39.4)	179 (32.2)	325 (41.3)	371 (42.2)	
Current	255 (11.5)	103 (18.5)	83 (10.5)	69 (7.8)	
**Alcohol drinking, *n* (%)**	1546 (69.6)	404 (72.7)	575 (73.1)	567 (64.5)	<.001
**Diabetes, *n* (%)**	526 (23.7)	85 (15.3)	154 (19.6)	287 (32.7)	<.001
**Coronary heart disease, *n* (%)**	212 (9.5)	53 (9.5)	76 (9.7)	83 (9.4)	.989
**Stroke, *n* (%)**	147 (6.6)	41 (7.4)	45 (5.7)	61 (6.9)	.429
**Hypertension, *n* (%)**	1390 (62.6)	268 (48.2)	483 (61.4)	639 (72.7)	<.001
**Hyperlipidemia, *n* (%)**	1283 (57.7)	268 (48.2)	480 (61.0)	535 (60.9)	<.001
**BMI, Mean ± SD, kg/m^2^**	29.4 ± 6.3	22.8 ± 1.7	27.4 ± 1.4	35.4 ± 5.2	<.001
**Waist, Mean ± SD, cm**	102.7 ± 14.3	87.6 ± 7.4	99.8 ± 7.0	115.0 ± 11.8	<.001

Abbreviations: BMI, body mass index; HEI, healthy eating index; PIR, poverty income ratio; SD, standard deviation.

The mean age of the participants was 69.3 ± 6.7 years old. Of the total, 51.8% of participants were female. Obese subjects were less likely to have graduated from college. Significant differences among normal, overweight, and obese subjects existed in age, sex, education level, race, alcohol drinking, smoking situation, and other characteristics. PSM analysis was conducted by correcting differences such as age, sex, education level, smoking status, and alcohol consumption, which are confounding factors affecting cognitive function. The baseline characteristics after PSM are shown in Table 2. After PSM, there was still a significant difference in the aspects of diabetes, hypertension, and hyperlipidemia. Obese people were more likely to suffer from chronic metabolic disease. The remaining characteristics did not differ among the three groups.

**TABLE 2:** Basic characteristics of participants after propensity score matching (PSM) analysis in the National Health and Nutrition Examination Survey (NHANES) 2011–2014.

Characteristic	Total (*n *= 834)	Normal (*n *= 278)	Overweight (*n *= 278)	Obese (*n *= 278)	*p* Value
**Age, Mean ± SD, years**	70.4 ± 6.9	70.2 ± 6.9	70.7 ± 6.9	70.1 ± 6.9	.594
**Sex, *n* (%)**					.794
Male	390 (46.8)	130 (46.8)	126 (45.3)	134 (48.2)	
Female	444 (53.2)	148 (53.2)	152 (54.7)	144 (51.8)	
**Race, *n* (%)**					.506
Mexican American	54 (6.5)	15 (5.4)	15 (5.4)	24 (8.6)	
Other Hispanic	75 (9.0)	28 (10.1)	21 (7.6)	26 (9.4)	
Non‐Hispanic White	527 (63.2)	171 (61.5)	188 (67.6)	168 (60.4)	
Non‐Hispanic Black	146 (17.5)	53 (19.1)	45 (16.2)	48 (17.3)	
Non‐Hispanic Asian	18 (2.2)	6 (2.2)	7 (2.5)	5 (1.8)	
Other/multiracial	14 (1.7)	5 (1.8)	2 (0.7)	7 (2.5)	
**Education level, *n* (%)**					.665
Less than 9th grade	60 (7.2)	20 (7.2)	17 (6.1)	23 (8.3)	
9–11th grade (12th grade with no diploma)	96 (11.5)	30 (10.8)	28 (10.1)	38 (13.7)	
High school graduate/GED	200 (24.0)	66 (23.7)	68 (24.5)	66 (23.7)	
Some college or AA degree	264 (31.7)	89 (32.0)	98 (35.3)	77 (27.7)	
College graduate or above	214 (25.7)	73 (26.3)	67 (24.1)	74 (26.6)	
**PIR, Mean ± SD**	2.7 ± 1.6	2.8 ± 1.6	2.7 ± 1.6	2.6 ± 1.6	.127
**Smoke, *n* (%)**					.770
Never	422 (50.6)	146 (52.5)	139 (50.0)	137 (49.3)	
Former	342 (41.0)	109 (39.2)	119 (42.8)	114 (41.0)	
Current	70 (8.4)	23 (8.3)	20 (7.2)	27 (9.7)	
**Alcohol drinking, *n* (%)**	601 (72.1)	199 (71.6)	204 (73.4)	198 (71.2)	.831
**Diabetes, *n* (%)**	186 (22.3)	42 (15.1)	52 (18.7)	92 (33.1)	<.001^b^, ^c^
**Coronary heart disease**, ***n* (%)**	81 (9.7)	28 (10.1)	25 (9.0)	28 (10.1)	.884
**Stroke**, ***n* (%)**	53 (6.4)	16 (5.8)	16 (5.8)	21 (7.6)	.604
**Hypertension**, ***n* (%)**	491 (58.9)	123 (44.2)	169 (60.8)	199 (71.6)	<.001^[Link]^, ^b^, ^c^
**Hyperlipidemia**, ***n* (%)**	459 (55.0)	134 (48.2)	157 (56.5)	168 (60.4)	.013^c^
**BMI, Mean ± SD**	28.3 ± 5.6	22.9 ± 1.6	27.4 ± 1.3	34.7 ± 4.5	<.001^[Link]^, ^b^, ^c^
**Waist, Mean ± SD**	100.6 ± 14.0	88.0 ± 7.4	99.3 ± 7.0	114.9 ± 11.2	<.001^[Link]^, ^b^, ^c^
**Physical activity, M (Q1, Q3), MET‐minute/week**	600 (0, 2145)	970 (0, 2520)	720 (0, 2270)	240 (0, 1530)	<.001^b^, ^c^
**Sleep score, M (Q1, Q3)**	1.0 (1.0, 2.0)	2.0 (1.0, 2.0)	1.0 (1.0, 2.0)	1.0 (1.0, 2.0)	.201
**HEI2020, Mean ± SD**	55.8 ± 11.9	57.4 ± 12.5	56.0 ± 10.8	53.9 ± 11.9	.002^b^

Abbreviations: BMI, body mass index; HEI, healthy eating index; MET, metabolic equivalent; PIR, poverty income ratio; SD, standard deviation.

^a^ There were differences between the normal and the overweight groups.

^b^ There were differences between the normal and the obese groups.

^c^ There were differences between the overweight and the obese groups.

### 3.2 Cognitive function and obesity index

**FIGURE 2:**
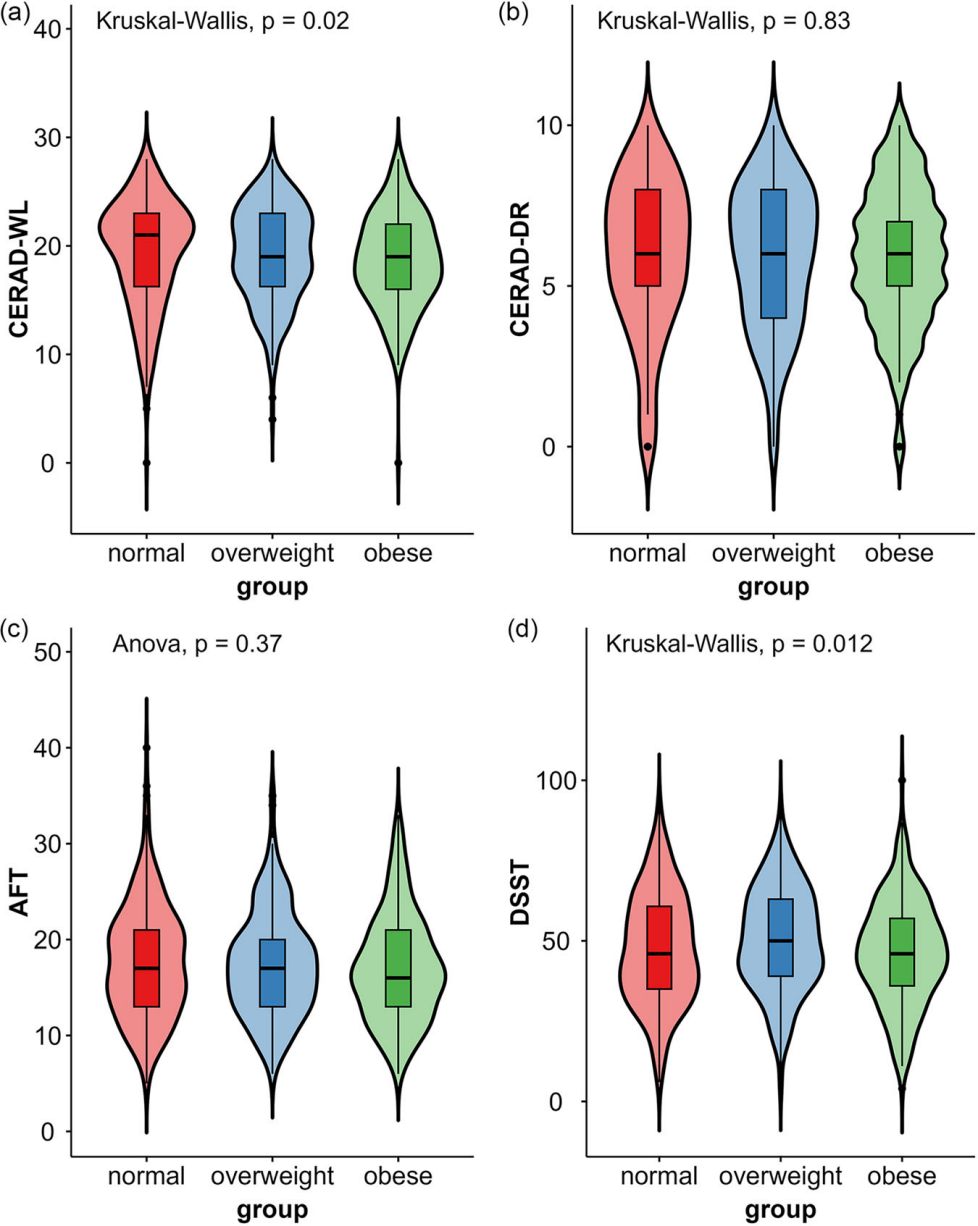
Cognitive function among the normal, overweight, and obese groups. Figures (a)‐(d) display the performance of participants in the cognitive assessments of CERAD‐WL, CERAD‐DR, AFT, and DSST, respectively.

**FIGURE 3:**
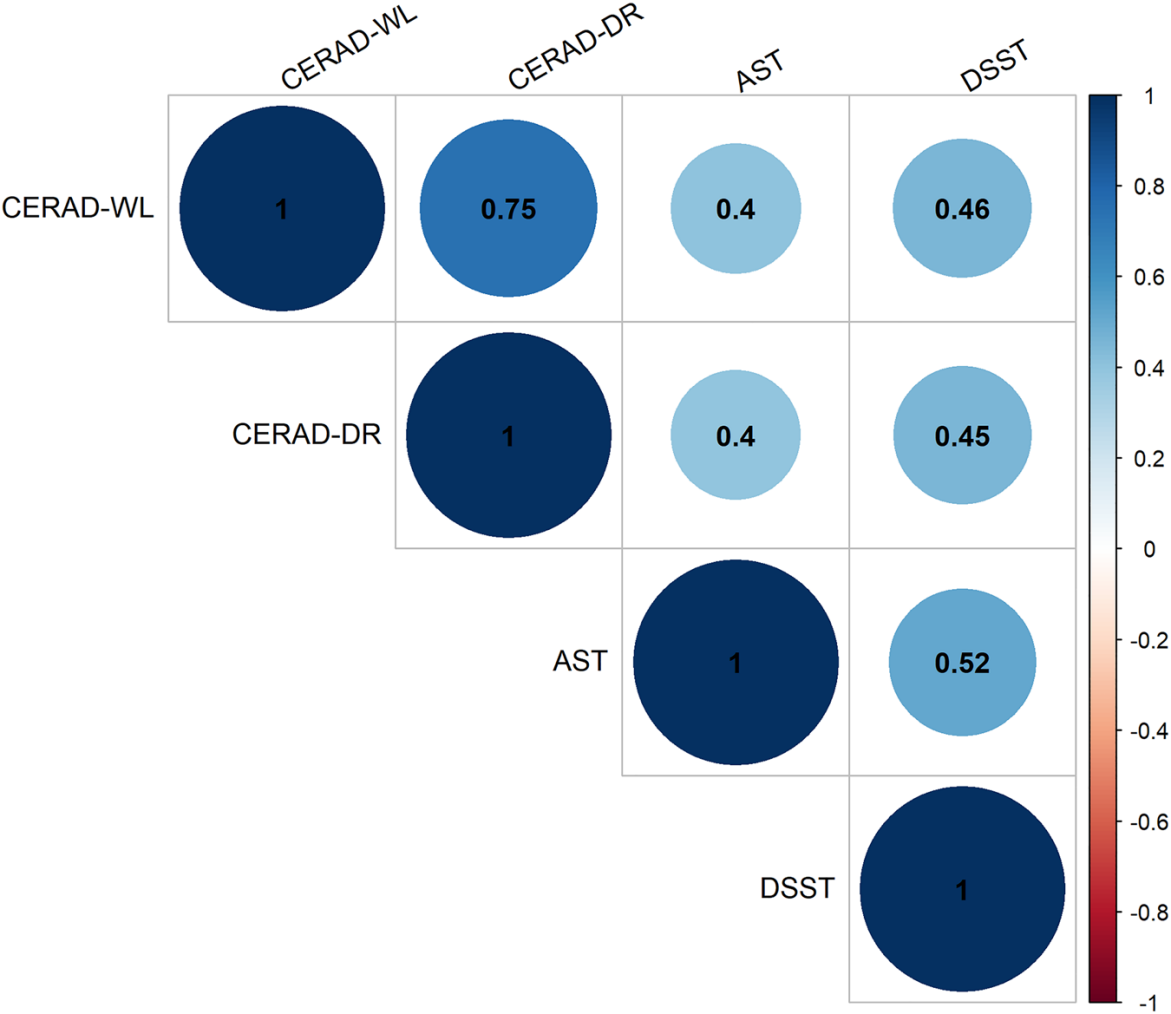
Heatmap of correlations between cognitive tests. The heatmap shows the correlations of all the cognitive tests. The numbers in circles represent the Spearman correlation coefficients.

Four cognitive assessments were tested in the NHANES. The cognitive performance of the 834 participants is shown in Table 3 and Figure 2. There were significant differences among the three groups in terms of the CERAD‐WL (*χ* ^2 ^= 7.804, *p* = .020) and DSST (*χ* ^2 ^= 8.869, *p* = .012) scores, which reflect learning ability and processing speed, respectively. In the CERAD‐WL test, overweight and obese people performed worse than normal‐weight seniors did in three learning trials, but only the difference between the normal and the obese groups was statistically significant. The obese group had lower DSST scores than did the overweight group. The overweight group performed better than the other two groups in the DSST. There was no significant difference in the CERAD delayed recall score or the AFT score. However, correlation analysis found statistically significant correlations between any two tests (*p *< .001). Figure 3 portrays the correlation of all the cognitive tests.

**TABLE 3:** Cognitive function among normal, overweight, and obesity groups.

	Total (*n* = 834)	Normal (*n *= 278)	Overweight (*n *= 278)	Obese (*n *= 278)	*F*/*χ* ^2^	*p* Value
**CERAD‐DR, M (Q_1_, Q_3_)**	6.0 (4.0, 8.0)	6.0 (5.0, 8.0)	6.0 (4.0, 8.0)	6.0 (5.0, 7.0)	*χ* ^2 ^= .377^a^	.828
**CERAD‐WL, M (Q1, Q3)**	19.0 (16.0, 22.0)	21.0 (16.2, 23.0)	19.0 (16.2, 23.0)	19.0 (16.0, 22.0)	*χ* ^2 ^= 7.804^a^	.020^c^ ^,b^
**AFT, mean ± SD**	17.4 ± 5.6	17.7 ± 5.9	17.4 ± 5.4	17.0 ± 5.5	*F *= .999^b^	.369
DSST**, M (Q_1_, Q_3_)**	47.0 (37.0, 60.0)	46.0 (35.0, 60.8)	50.0 (39.0, 63.0)	46.0 (36.0, 57.0)	*χ* ^2 ^= 8.869^a^	.012^c^ ^,c^

*Note*: CERAD‐WL: Total Score (3 Recall trials) of CERAD; CERAD‐DR: Delayed Recall Score of CERAD.

Abbreviations: AFT, animal fluency test; CERAD, Consortium to Establish a Registry for Alzheimer's Disease; DSST, digit symbol substitution test; SD, standard deviation.

^a^ Kruskal–Wallis *H* test.

^b^ One Way ANOVA.

^c^ *p* < .05.

### 3.3 Results of multiple regression models

We further explored the relationships between obesity and cognitive function, regarding obesity indicators as continuous measures. We constructed three multivariable linear regression models (Table 4): Model 1, no covariates were adjusted; Model 2, age, sex, race, and education level were adjusted; Model 3, age, sex, race, education level, PIR, smoking status, drinking, diabetes, hyperlipidemia, hypertension, stroke history, and coronary artery disease were adjusted. Table 4 shows that BMI was negatively associated with the scores of CERAD‐WL (*β* = −.098, *p* = .008), AFT (*β* = −.115, *p* = .037), and DSST (*β* = −.380, *p* = .029). After controlling for basic covariates (Model 2), a higher BMI still showed an inverse association with the CERAD‐WL and DSST scores. In addition, the association between BMI and AFT after controlling for age, sex, race, and education level was marginally significant (*p* = .051). Similar to the BMI results, WC showed a negative association with the scores of CERAD‐WL, AFT, and DSST. It is worth noting that this negative association still existed between WC and CERAD‐WL scores in our fully adjusted Model 3 (*β* = −.029, *p* = .045). In addition to common influencing factors, such as age, sex, race, and education, we additionally adjusted for BMI and found that WC was still negatively correlated with the CERAD‐WL (*β* = −.083, *p* = .022) and DSST (*β* = −.319, *p* = .009).

**TABLE 4:** Linear regression model for body mass index (BMI) and waist circumference on different test scores of cognitive functions in the elderly people.

	Model 1			Model 2			Model 3		
	*β*	SE	*p*	*β*	SE	*p*	*β*	SE	*P*
***BMI***									
CERAD‐WL	−.098	.035	.008^a^	−.080	.024	.003^a^	−.042	.023	.094
CERAD‐DR	−.014	.016	.380	−.008	.012	.540	.011	.015	.469
AFT	−.115	.053	.037^a^	−.083	.040	.051	−.027	.044	.556
DSST	−.380	.167	.029^a^	−.251	.107	.030^a^	−.069	.089	.457
**WC**									
CERAD‐WL	−.060	.014	*p* < .001^a^	−.044	.011	*p* < .001	−.029	.013	.045^a^
CERAD‐DR	−.013	.007	.062	−.004	.005	.417	.004	.006	.556
AFT	−.040	.020	.047^a^	−.037	.018	.049^a^	−.012	.019	.529
DSST	−.192	.072	.012^a^	−.096	.046	.048^a^	−.034	.042	.426
**BMI‐weighted WC**									
CERAD‐WL	−.123	.023	*p* < .001^a^	−.083	.033	.022^a^	−.069	.038	.102
CERAD‐DR	−.050	.011	*p* < .001^a^	−.022	.012	.082	−.014	.013	.302
AFT	−.036	.029	.226	−.070	.041	.099	−.048	.045	.308
DSST	−.422	.116	.001^a^	−.319	.110	.009^a^	−.218	.116	.089

*Note*: CERAD‐WL: Total Score (3 Recall trials) of CERAD; CERAD‐DR: Delayed Recall Score of CERAD. Model 1: No covariates were adjusted. Model 2: Age, sex, race, and education were adjusted. Model 3: Age, sex, race, education, PIR, smoking status, drinking, diabetes, hyperlipidemia, hypertension, stroke history, and coronary artery disease were adjusted.

Abbreviations: AFT, animal fluency test; CERAD, Consortium to Establish a Registry for Alzheimer's Disease; DSST, digit symbol substitution test; WC, waist circumference.

^a^ *p* < .05

**p < .01

***p < .001.

### 3.4 Partial least squares structural equation modeling

**FIGURE 4:**
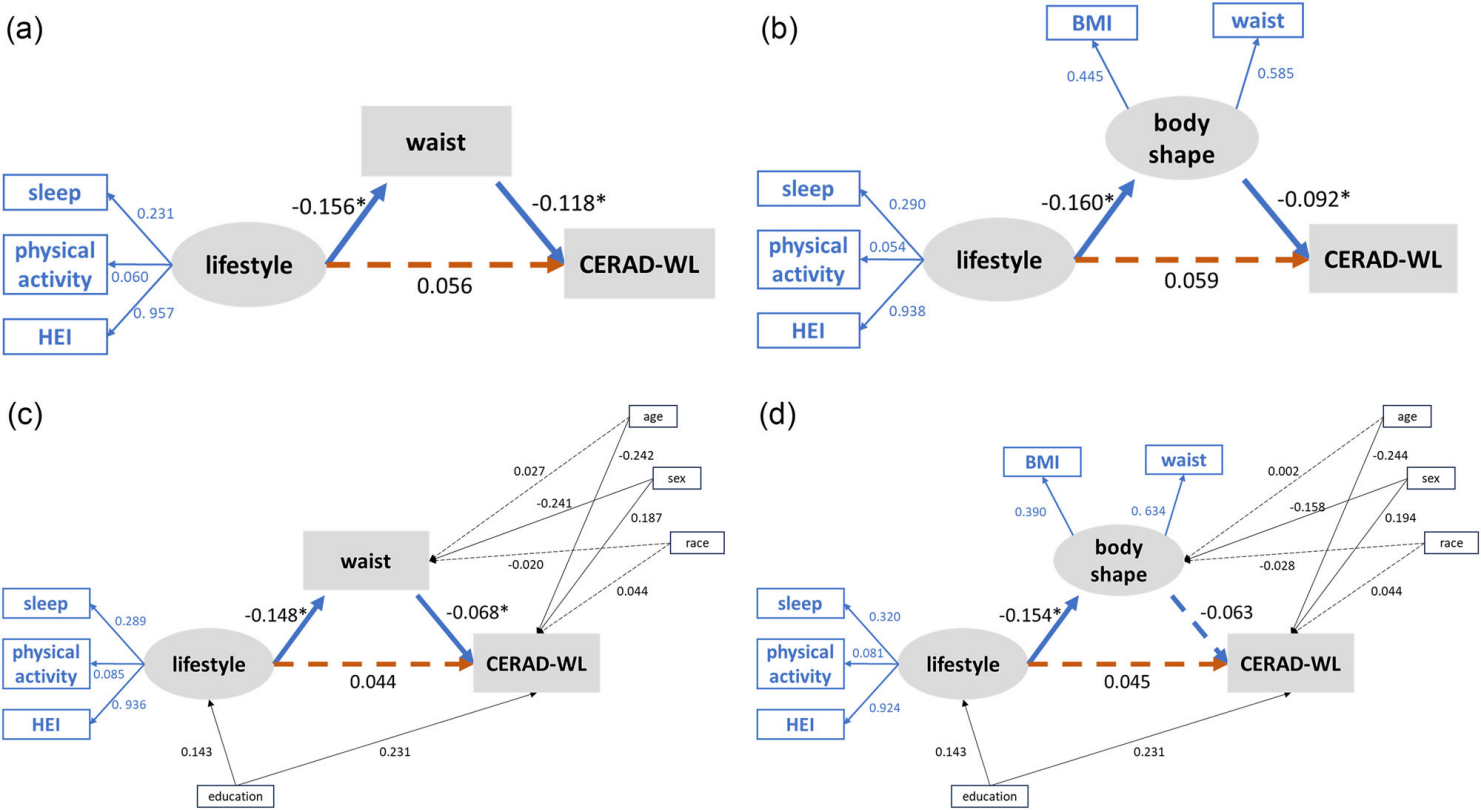
The partial least squares structural equation model (PLS‐SEM) of the effects of lifestyle and body shape on Consortium to Establish a Registry for Alzheimer's Disease (CERAD)‐WL scores. (a) The SEM is constructed based on regression results, illustrating the relationship between lifestyle, waist circumference (WC), and cognition. Lifestyle is a latent variable, which is indicated by sleep, physical activity, and Healthy Eating Index (HEI)‐2020. (b) This model replaces the mediator variable "WC" with a latent variable. Body shape, which is indicated by body mass index (BMI) and waist circumference, is another latent variable in models (b) and (d). Figures (c) and (d) show the modified models that control for variables such as age, sex, race, and education level, based on the frameworks established in (a) and (b), respectively. Negative and positive effects are indicated by dashed and solid lines, respectively. The blue number on the blue line represents the weight of each observable variable over the latent variable. The black numbers represent the coefficients of each path. In (d), the “body shape to CERAD‐WL” path is marginally significant (*p* = .059). (When setting the test level *α* at .05, caution must be exercised in interpreting *p*‐values that are close to the critical threshold, as negative results may stem from sampling errors. Such scenarios are commonly described as partially significant or marginally significant, for which the statistical result often contains *p* values between .05 and .10.)

PLS‐SEM was used to assess the impact of obesity on cognitive function. Figure 4 shows the full constructs of PSL‐SEMs and the parameter estimates for the final models. To explore the meaning of weight management in real life, we started with a theoretical model based on our hypothesis that body shape (BMI or WC) would mediate the association between one's lifestyle and cognitive function. According to the results of the regression model, we constructed a model using WC for body shape and the CERAD‐WL score for cognitive function. As shown in Figure 4a, the results indicated that there were no significant direct paths from lifestyle to CERAD‐WL (*β* = .056, *p* = .115). Lifestyle, the exogenous latent variable in this model, significantly affected WC (*β* = −.156, *p* < .001), and WC significantly negatively correlated with CERAD‐WL score (*β* = −.118, *p* < .001). Furthermore, the Sobel test was performed to assess the significance of the model's indirect effects (Table S3). Waist status completely mediated the path from lifestyle to CERAD‐WL, with a mediating effect of 0.018 (*p* for Sobel test = .008). We then performed sensitivity analyses. First, we replaced the WC with a latent variable that was measured by BMI and WC (Figure 4b). Subsequently, we included age, sex, race, and education in the model as control variables. The results resembled our first SEM (Figure 4c,d). The path from lifestyle to CERAD‐WL was mediated considerably by waist or body shape indicators. In addition, we constructed a latent variable called “cognitive function,” which is formed by four cognitive tests. The latent variable “cognitive function” replaced the position of “CERAD‐WL” in the present model. However, the new model is not significant.

## 4 DISCUSSION

This cross‐sectional study was designed to investigate the patterns of how obesity affects cognitive function. Using a sample from older adults in the United States, we observed that obese individuals had lower CERAD‐WL scores, which reflected worse verbal learning ability and episodic memory. After fully adjusting for confounding factors that may influence cognitive function, higher WC was associated with lower cognitive function, as evaluated through the CERAD‐WL. There were also negative associations between higher BMI and lower cognition, as well as between higher WC and lower cognition in the AFT and DSST. Finally, we found that WC played a mediating role in the lifestyle–cognitive functional pathway. This present study indicated that obese older adults showed decreases in cognitive functions, such as learning ability, episodic memory, language, and executive function, most of which were domains measured by the CERAD test. The relationship also has important implications in real life, because daily lifestyle, such as sleep, diet, and PA, reflects cognitive performance through weight management.

These findings are consistent with our hypotheses, aligning with previous studies of obesity's association with cognitive function (Quaye et al., 2023; Tanaka et al., 2020). In a large study consisting of 28,867 participants in America, obese participants had lower baseline global cognition than normal‐weight participants (Quaye et al., 2023). Relevant studies have documented an inverse correlation among different aspects of cognitive function and various anthropometric measures of obesity, such as BMI, WC, and other anthropometric measures (Li et al., 2022). Additionally, poorer global cognitive function was observed with increasing age in patients with larger WC and WHR (Dye et al., 2017). Among various cognitive domains, executive function, WM, and verbal learning are often the most affected cognitive domains in obese individuals (Dye et al., 2017; Tanaka et al., 2020). Our results also confirm this. In this study, impaired WM performance was also demonstrated in obese old adults compared with healthy weight controls. Preclinical and clinical AD is also characterized by deficits in episodic memory, WM, and executive function (Kirova et al., 2015; Zokaei & Husain, 2019). Obesity‐affected brain areas overlap with AD‐vulnerable brain regions, following the same cortical thinning pattern with the temporoparietal and frontal lobes as the first impaired cortex (Morys et al., 2023). Obesity and AD exhibit comparable cognitive performance and brain structure, which further indicates that obesity is a risk factor for dementia (Floud et al., 2020).

In this study, higher WC was associated with lower CERAD‐WL scores even after adjusting for economic factors, smoking status, alcohol consumption, diabetes, hypertension, and other confounding factors. However, in the same model of full adjustment, there was no significant relationship between BMI and CERAD‐WL scores. Because WC reflects abdominal obesity more than BMI, we believe that adipose tissue distribution, namely, the type of obesity, also impacts cognitive function. A larger WC was associated with a greater rate of cognitive decline. These results are supported by several studies showing that abdominal obesity is a more salient predictor of cognitive decline than whole‐body adiposity (Kerwin et al., 2011; Tanaka et al., 2020). The results of a cohort study are consistent with our finding that a greater WC is associated with a greater rate of cognitive decline, whereas BMI does not have such a relationship (West et al., 2017).

Sleep, PA, and diet are the three major behavioral factors affecting body weight, and lifestyle intervention serves as an important measure in obesity treatment (Blüher, 2019; Wadden et al., 2020). This study showed that WC can completely mediate the relationship between lifestyle and cognitive function. According to our model, a healthier lifestyle predicts lower markers of obesity, which further predicts better cognitive function. Associations between lifestyle factors and cognition have been previously demonstrated (De Sousa et al., 2021; Hoscheidt et al., 2022; Paller et al., 2021; Więckowska‐Gacek et al., 2021; Yang et al., 2023). These modifiable lifestyle factors may supply cognitive reserve, which is the ability to cope with brain pathological changes and attenuate cognitive decline (Song et al., 2022). Healthy eating habits could increase cerebral perfusion and reduce the accumulation of AD biomarkers in adults with normal cognition (Hoscheidt et al., 2022). A healthy diet features a high intake of vegetables, fresh fruits, and seafood, the beneficial effects of which on cognition might be attributed to anti‐oxidant, anti‐inflammatory, and anti‐diabetic effects and enough mono‐/poly‐unsaturated fats (Więckowska‐Gacek et al., 2021; Zhang et al., 2021). Our study revealed that sleep problems are a pivotal life factor contributing to weight gain and, subsequently, affect cognitive function. Notably, sleep disturbance is a significant risk factor for cognitive impairment. Individuals with sleep disorders are more likely to experience decreased metabolite clearance and increased accumulation of neurotoxic substances (Shi et al., 2018). Moreover, inadequate sleep and other disturbances are important metabolic stressors, which stimulate appetite and reduce 24‐h energy expenditure (Chaput et al., 2023). PA seems to be an excellent intervention for preventing cognitive impairment in obese patients (De Sousa et al., 2021). Falck et al. (2018) reported that more frequent PA in older adults is associated with better global cognition. Obese people can also benefit from physical exercise. A morning bout of moderate‐intensity exercise improved WM or executive function in overweight/obese older patients (Wheeler et al., 2020). The cognitive benefits of regular and proper PA include restoring insulin sensitivity (Kullmann et al., 2022), attenuating neuroinflammation, and increasing cerebral blood flow (De La Rosa et al., 2020).

This research was founded on statistics from the NHANES, which features a complex, multistage, and probability sampling design. All results, including the PLS‐SEM analysis, were based on the data of correct NHANES sampling weights, making the research samples more typical. In addition, the researchers controlled for potential confounding variables in order to increase the credibility of the results, both in multivariable linear regression analysis and PLS‐SEM analysis. Furthermore, we used PLS‐SEM rather than traditional covariance‐based SEM, because PLS has no data distribution requirements and can be applied to stratified sampling data (Sarimanah et al., 2022). However, the current research still has some limitations. The cross‐sectional nature of the study is a limitation in the interpretation of our findings, so we could not determine a causal relationship between the obesity index and cognition. Moreover, we did not include physiological parameters in our SEM, which might be helpful for explaining the mechanism underlying the relationships among lifestyle, obesity, and cognition. Furthermore, data on cognitive function were only available in the NHANES for the years 2011–2014, and these cognitive data were limited to just three cognitive tests. This prevents us from exploring the effects of obesity on other important and vulnerable cognitive domains, including visuospatial ability and processing speed. Finally, because the NHANES included only limited tests, we could only infer the impact of obesity on a certain cognitive domain from a single‐observed test score.

In summary, this study comprehensively investigated the relationships between obesity measures and cognition in older Americans. We mainly found that obesity impaired learning ability and WM. Simultaneously, we demonstrated that the effects of lifestyle on cognition were mediated by obesity‐related anthropometric indices. Sleep, PA, and diet influenced the degree of obesity, which in turn determined cognition. Aggressive weight management and maintaining a healthy lifestyle might prevent cognitive decline.

## AUTHOR CONTRIBUTIONS

**Leian Chen**: Conceptualization; methodology; formal analysis; writing—original draft; visualization. **Yu Sun** and **Dantao Peng**: Writing—review and editing; supervision. **Ying Hou**: Validation; formal analysis.

## CONFLICT OF INTEREST STATEMENT

The authors have no conflicts of interest to report.

## FUNDING INFORMATION

The National High Level Hospital Clinical Research Funding; The Elite Medical Professionals Project of China‐Japan Friendship Hospital, Grant No. ZRJY2021‐QM07; The National Natural Science Foundation of China, Grant No. 82201570; Central health research project, Grant No. 2020ZD10

### PEER REVIEW

The peer review history for this article is available at https://publons.com/publon/10.1002/brb3.70006.

## Supporting information

Table S1 Healthy Eating Index (HEI) 2020 components and scoring standards.
Table S2 Survey‐weighted characteristics of the total sample.
Table S3 Sobel test results for indirect effects of lifestyle, waist, and CERAD‐WL.

## Data Availability

The data supporting the findings of this study are openly available in NHANES database at https://wwwn.cdc.gov/nchs/nhanes/.
