# Supplementary material for: Association of obesity indicators with cognitive function among US adults aged 60 years and older: Results from NHANES
Source: Brain Behav. 2024 Sep 11;14(9):e70006. doi: 10.1002/brb3.70006 (PMC11391027; doi:10.1002/brb3.70006)
Supplement: Supplementary file 1 — Table S1 Healthy Eating Index (HEI) 2020 components and scoring standards. Table S2 Survey‐weighted characteristics of the total sample. Table S3 Sobel test results for indirect effects of lifestyle, waist, and CERAD‐WL. [file BRB3-14-e70006-s001.docx]

Supplement

Table S1. Healthy Eating Index (HEI) 2020 components and scoring standards

| **Healthy Eating Index-2020 (Applies to Ages 2 y and Older)^a^** | | | |
| --- | --- | --- | --- |
| **Component** | **Maximum points** | **Standard for maximum score** | **Standard for minimum score of 0** |
| **Adequacy components** |  |  |  |
| Total Fruits^[b](https://www.sciencedirect.com/science/article/pii/S2212267223002460?via%3Dihub#tbl1fnb)^ | 5 | ≥0.8 cup eq/1,000 kcal | No Fruit |
| Whole Fruits^c^ | 5 | ≥0.4 cup eq/1,000 kcal | No Whole Fruit |
| Total Vegetables^d^ | 5 | ≥1.1 cup eq/1,000 kcal | No Vegetables |
| Greens and Beans^d^ | 5 | ≥0.2 cup eq/1,000 kcal | No Dark Green Vegetables or Legumes |
| Whole Grains | 10 | ≥1.5 oz eq/1,000 kcal | No Whole Grains |
| Dairy^e^ | 10 | ≥1.3 cup eq/1,000 kcal | No Dairy |
| Total Protein Foods^d^ | 5 | ≥2.5 oz eq/1,000 kcal | No Protein Foods |
| Seafood and Plant Proteins^f^ | 5 | ≥0.8 oz eq/1,000 kcal | No Seafood or Plant Proteins |
| Fatty Acids^g^ | 10 | (PUFAs^h^ + MUFAs^[i](https://www.sciencedirect.com/science/article/pii/S2212267223002460?via%3Dihub#tbl1fni)^) / SFAs^[j](https://www.sciencedirect.com/science/article/pii/S2212267223002460?via%3Dihub#tbl1fnj)^ ≥2.5 | (PUFAs + MUFAs) / SFAs ≤1.2 |
| **Moderation components** |  |  |  |
| Refined Grains | 10 | ≤1.8 oz eq/1,000 kcal | ≥4.3 oz eq/1,000 kcal |
| Sodium | 10 | ≤1.1 g/1,000 kcal | ≥2.0 g/1,000 kcal |
| Added Sugars | 10 | ≤6.5% of energy | ≥26% of energy |
| Saturated Fats | 10 | ≤8% of energy | ≥16% of energy |

a. The Healthy Eating Index-2020 components and scoring standards are the same as the Healthy Eating Index-2015. Intakes between the minimum and maximum standards are scored proportionately. Component scores are summed to create the total score.

b. Includes 100% fruit juice.

c. Includes all forms except juice.

d. Includes beans, peas, and lentils.

e. Includes all milk products, such as fluid milk, yogurt, and cheese, and fortified soy beverages.

f. Includes seafood, nuts, seeds, [soy products](https://www.sciencedirect.com/topics/agricultural-and-biological-sciences/soy-product) (other than beverages), and beans, peas, and lentils.

g. Ratio of poly- and [monounsaturated fatty acids](https://www.sciencedirect.com/topics/medicine-and-dentistry/monounsaturated-fatty-acid) (PUFAs and MUFAs) to saturated fatty acids (SFAs).

h. PUFA = polyunsaturated fatty acid.

i. MUFA = monounsaturated fatty acid.

j. SFA = saturated fatty acid.

The HEI-2020 contains 13 components that sum to a total maximum score of 100 points. The total score is the sum of the score of adequacy components (i.e. foods to eat more of for good health) and moderation components (i.e. foods to limit for good health). Nine of these components evaluate adequacy (total fruit, whole fruit, total vegetables, greens and beans, whole grains, dairy, total protein foods, seafood and plant proteins and fatty acids) and four of these components evaluate moderation (refined grains, sodium, added sugars and saturated fat).

Table S2. Survey-weighted characteristics of the total sample

| **Characteristic** | **normal** | **overweight** | **obesity** | ***P* value** |
| --- | --- | --- | --- | --- |
|  | **(*n*=556)** | **(*n*=787)** | **(*n*=879)** |  |
| **Age, Mean±SD,years** | 70.1±6.8 | 69.8±6.9 | 68.3±6.4 | <0.001 |
| **Sex, *n* (%)** |  |  |  | <0.001 |
| Male | 264 (47.5) | 441 (56.0) | 365 (41.5) |  |
| Female | 292 (52.5) | 346 (44.0) | 514 (58.5) |  |
| **Ethnicity, *n* (%)** |  |  |  | <0.001 |
| Mexican American | 28 (5.0) | 71 (9.0) | 85 (9.7) |  |
| Other Hispanic | 44 (7.9) | 86 (10.9) | 77 (8.8) |  |
| Non-Hispanic White | 288 (51.8) | 427 (54.3) | 426 (48.5) |  |
| Non-Hispanic Black | 106 (19.1) | 143 (18.2) | 267 (30.4) |  |
| Non-Hispanic Asian | 82 (14.7) | 49 (6.2) | 13 (1.5) |  |
| Other Race / Multi-Racial | 8 (1.4) | 11 (1.4) | 11 (1.3) |  |
| **Education level, *n* (%)** |  |  |  | 0.002 |
| Less than 9th grade | 56 (10.1) | 66 (8.4) | 87 (9.9) |  |
| 9-11th grade (12th grade with no diploma) | 62 (11.2) | 106 (13.5) | 124 (14.1) |  |
| High school graduate/GED | 124 (22.3) | 183 (23.3) | 217 (24.7) |  |
| Some college or AA degree | 143 (25.7) | 227 (28.8) | 286 (32.5) |  |
| College graduate or above | 171 (30.8) | 205 (26.0) | 165 (18.8) |  |
| **PIR, Mean±SD** | 2.8±1.6 | 2.8±1.6 | 2.5±1.6 | 0.006 |
| **Smoke, *n* (%)** |  |  |  | <0.001 |
| Never | 274 (49.3) | 379 (48.2) | 439 (49.9) |  |
| Former | 179 (32.2) | 325 (41.3) | 371 (42.2) |  |
| Current | 103 (18.5) | 83 (10.5) | 69 (7.8) |  |
| **Alcohol drinking, *n* (%)** | 404 (72.7) | 575 (73.1) | 567 (64.5) | <0.001 |
| **Diabetes, *n* (%)** | 85 (15.3) | 154 (19.6) | 287 (32.7) | <0.001 |
| **Coronary heart disease**, *n* (%) | 53 (9.5) | 76 (9.7) | 83 (9.4) | 0.989 |
| **Stroke**, *n* (%) | 41 (7.4) | 45 (5.7) | 61 (6.9) | 0.429 |
| **Hypertension**, *n* (%) | 268 (48.2) | 483 (61.4) | 639 (72.7) | <0.001 |
| **Hyperlipidemia**, *n* (%) | 268 (48.2) | 480 (61.0) | 535 (60.9) | <0.001 |
| **BMI, Mean±SD** | 22.8±1.7 | 27.4±1.4 | 35.4±5.2 | <0.001 |
| **WAIST, Mean±SD** | 87.6±7.4 | 99.8±7.0 | 115.0±11.8 | <0.001 |
| **Characteristic** | **normal** | **overweight** | **obesity** | ***P* value** |
|  | **(N = 10,797,334)** | **(N = 15,723,300)** | **(N = 16,595,174)** |  |
| **Age, Mean±SD,years** | 69.6±6.7 | 69.3±6.7 | 68.0±6.3 | <0.001 |
| **Sex, *n* (%)** |  |  |  | 0.002 |
| Male | 4,464,704 (41.4) | 8,267,926 (52.6) | 7,033,199 (42.4) |  |
| Female | 6,332,630 (58.6) | 7,455,374 (47.4) | 9,561,975 (57.6) |  |
| **Ethnicity, *n* (%)** |  |  |  | <0.001 |
| Mexican American | 183,240 (1.7) | 526,471 (3.3) | 587,884 (3.5) |  |
| Other Hispanic | 267,520 (2.5) | 578,890 (3.7) | 531,280 (3.2) |  |
| Non-Hispanic White | 8,857,173 (82.0) | 13,157,957 (83.7) | 13,342,615 (80.4) | |
| Non-Hispanic Black | 646,659 (6.0) | 903,074 (5.7) | 1,775,314 (10.7) | |
| Non-Hispanic Asian | 640,053 (5.9) | 340,710 (2.2) | 108,717 (0.7) |  |
| Other/Multi-Racial | 202,689 (1.9) | 216,198 (1.4) | 249,364 (1.5) |  |
| **Education level, *n* (%)** |  |  |  | 0.055 |
| Less than 9th grade | 527,259 (4.9) | 771,757 (4.9) | 813,218 (4.9) |  |
| 9-11th grade (12th grade with no diploma) | 857,748 (7.9) | 1,481,703 (9.4) | 1,841,232 (11.1) | |
| High school graduate/GED | 2,387,040 (22.1) | 3,395,311 (21.6) | 3,540,053 (21.3) | |
| Some college or AA degree | 2,986,128 (27.7) | 4,737,913 (30.1) | 6,238,532 (37.6) | |
| College graduate or above | 4,039,158 (37.4) | 5,336,616 (33.9) | 4,162,139 (25.1) | |
| **PIR, Mean±SD** | 3.32±1.55 | 3.27±1.55 | 3.00±1.55 | 0.002 |
| **Smoke, *n* (%)** |  |  |  | <0.001 |
| Never | 5,308,369 (49.2) | 8,118,966 (51.6) | 7,651,582 (46.1) | |
| Former | 3,702,567 (34.3) | 6,320,927 (40.2) | 7,743,683 (46.7) | |
| Current | 1,786,398 (16.5) | 1,283,407 (8.2) | 1,199,908 (7.2) |  |
| **Alcohol drinking, *n* (%)** | 8,495,443 (78.7) | 11,773,965 (74.9) | 11,471,155 (69.1) | 0.001 |
| **Diabetes, *n* (%)** | 1,180,069 (10.9) | 2,388,624 (15.2) | 4,757,678 (28.7) | <0.001 |
| **Coronary heart disease**, *n* (%) | 905,886 (8.4) | 1,430,126 (9.1) | 1,853,285 (11.2) | 0.347 |
| **Stroke**, *n* (%) | 637,372 (5.9) | 809,302 (5.1) | 1,073,623 (6.5) | 0.65 |
| **Hypertension**, *n* (%) | 4,346,223 (40.3) | 9,012,135 (57.3) | 11,787,517 (71.0) | <0.001 |
| **Hyperlipidemia**, *n* (%) | 4,931,757 (45.7) | 9,876,852 (62.8) | 10,620,303 (64.0) | <0.001 |
| **BMI, Mean±SD** | 22.7±1.7 | 27.4±1.4 | 35.4±5.4 | <0.001 |
| **WAIST, Mean±SD** | 87.5±7.4 | 100.4±7.0 | 115.7±11.7 | <0.001 |

PIR, poverty income ratio;

BMI, body mass index;

Table S3. Sobel test results for indirect effects of lifestyle, waist and CERAD-WL

|  | **Independent variables** | **Mediating**  **variables** | **Dependent**  **variables** | *T-values* | *Std. error* | *p-value* |
| --- | --- | --- | --- | --- | --- | --- |
| Figure 4a | lifestyle | waist | CERAD-WL | 2.667 | 0.007 | 0.008** |
| Figure 4c | lifestyle | waist | CERAD-WL | 2.251 | 0.007 | 0.024* |

Std. Error, Standard Error.

*p < 0.05, **p<0.01
